# Comparison of Postoperative Outcomes Between Percutaneous Endoscopic Lumbar Interbody Fusion and Minimally Invasive Transforaminal Lumbar Interbody Fusion for Lumbar Spinal Stenosis

**DOI:** 10.3389/fsurg.2022.916087

**Published:** 2022-06-15

**Authors:** Lu Lin, Xiao-Qin Liu, Lei Shi, Si Cheng, Zhi-Qiang Wang, Qi-Jun Ge, Ding-Zhi Gao, Amadou Cheffou Ismail, Zhen-Yong Ke, Lei Chu

**Affiliations:** Department of Spine Surgery, The Second Affiliated Hospital of Chongqing Medical University, Chongqing, China

**Keywords:** lumbar spinal stenosis, postoperative outcomes, transforaminal lumbar interbody fusion, endoscopy, minimally invasive

## Abstract

**Objective:**

This study aimed to compare postoperative outcomes in surgical and patient-reported outcomes (PROs) between percutaneous endoscopic lumbar interbody fusion (PE-LIF) and minimally invasive transforaminal lumbar interbody fusion (MIS-TLIF) for the treatment of lumbar spinal stenosis (LSS).

**Methods:**

We reviewed a total of 89 patients undergoing single-level surgery for lumbar spinal stenosis from January 2018 to July 2021. The cases were categorized as PE-LIF (Group PE-LIF, 41 cases) or MIS-TLIF (Group MIS-TLIF, 48 cases) approach. Parameters obtained at baseline through at least six months of follow-up were collected. The surgical outcomes involving the operative time, estimated blood loss, postoperative bed staying time, and length of hospital stays were analyzed. PROs included the Visual Analogue Scale (VAS), Oswestry Disability Index (ODI), modified MacNab standard evaluation, intervertebral fusion rate, and postoperative complications.

**Results:**

A total of 89 patients were included in this analysis involving 41 patients who underwent PE-LIF and 48 patients who underwent MIS-TLIF. The 2 groups were similar in gender, age, body mass index, follow-up time and surgery levels (*P* > 0.05), and were not significantly different in the length of hospital stays (*P* > 0.05). PE-LIF had a significantly longer operative time, greater fluoroscopy time, lower estimated blood loss and shorter bed rest time than MIS-TLIF. Both groups improved significantly from baseline for the VAS and ODI scores. PE-LIF was associated with a lower VAS score for back pain at three-day after surgery. There were no significant differences between PE-LIF and MIS-TLIF in the excellent or good rates and intervertebral fusion rates at the last follow-up (*P* > 0.05). As for related complications, there were no significant complications occurred, and no significant differences were seen in the complications between both groups (*P* > 0.05).

**Conclusions:**

To summarize, PE-LIF and MIS-TLIF are both safe and effective for LSS. PE-LIF has a definite short-term curative effect with less trauma.

## Introduction

Lumbar spinal stenosis (LSS) is highly prevalent in patients older than 60 years of age and is one of the most common reasons for spinal surgery (1). The incidence of LSS is expected to grow further as the Chinese population ages. It is believed that LSS is a frequent cause of low back pain and neurogenic claudication and can dramatically decrease patient quality of life (2). LSS makes patients suffer from substantial pain and reduces physical activity, and potentially increases the risk of chronic diseases, including cardiovascular diseases and neurodegenerative diseases (3). Conservative management (therapeutic lifestyle changes, physiotherapy, rehabilitation training, drugs, and epidural steroid injection) is always recommended before symptoms worsen (4).

Surgical treatment is essential when conservative treatment fails. Surgical treatment of LSS aims to decompress neural structures, restore stability to the spine, relieve symptoms, and improve function (5). The posterior lumbar interbody fusion (PLIF) is considered the gold standard, performed well by most spinal surgeons. However, it may be limited by iatrogenic injury of posterior ligament complex, inadequate restore lordosis, and potential retraction injury of nerve roots (6). The transforaminal lumbar interbody fusion (TLIF), first reported by Harms and Rolinger and developed by Harms and Blumes, could result in lower structural damage than the PLIF procedure (7). So far, both PLIF and TLIF have been extensively accepted and successfully applied in the management of LSS. But some scholars still doubt these traditional operations by their much soft-tissue disruption and high complication rates (8). With the development of minimally invasive surgery (MIS), MIS-TLIF has been reported to be a safe procedure with satisfactory outcomes and acceptable complications when compared with TLIF (9). In recent years, spinal surgeons have shown increased interest in percutaneous endoscopic lumbar interbody fusion (PE-LIF). This procedure is performed under a working channel and endoscopic system, which theoretically achieves less surgical trauma (10).

Both PE-LIF and MIS-TLIF are derived from the theory of open LIF. Despite more consensus on the sufficient efficacy of these surgical techniques, relevant evidence is still insufficient. Therefore, we conducted the present study to demonstrate the efficacy and safety of PE-LIF compared with MIS-TLIF in the treatment of LSS. We also briefly describe the technical notes and notable matters of PE-LIF.

## Methods

### Patient Selection and Data Collection

The present study was approved by the Ethics Committee of the Second Affiliated Hospital of Chongqing Medical University. All patients had signed a written informed consent before surgery. We retrospectively collected the clinical data of patients with LSS who underwent PE-LIF or MIS-TLIF by the same team of senior surgeons from January 2018 to July 2021. Relevant demographic information, clinical symptoms, and radiological outcomes were obtained.

The inclusion criteria were: (1) participants 45 years of age or older with a symptom of intermittent neurogenic claudication and at least one typical sign; (2) imaging indicating single-level lumbar central/lateral recess stenosis; (3) failing to relieve of symptoms after 4-6 weeks of conservative treatment; (4) at least six months of postoperative follow-up and complete patient-reported outcomes (PROs). The exclusion criteria included: (1) previous surgical history of the corresponding segment; (2) spinal trauma, infection, tuberculosis, tumor, and degenerative deformity.

### Surgical Technique

#### PE-LIF (L4/5 Segment)

After general anesthesia, the patient was positioned prone on the operating table with appropriate abdominal suspension. A C-arm fluoroscope was used to locate the surgical segment and marked the projection of the spinous process, intervertebral space, and pedicle. The puncture site was located at 2 cm lateral to the spinous process, and a 1.5 cm incision was made laterally on the significant symptom side. The puncture needle was placed at the posterior edge of the disc and vertebral body while it approached near the medial site of the articular process with the AP view of the C-arm fluoroscope. With the assistance of a puncture needle, the dilating cannulas were inserted progressively to establish a working cannula. A part of the facet joint and lamina was removed by the circular saw to enlarge the vision of the surgical field. Under direct endoscopic visualization, the nuclear material and proliferative ligamentum flavum were removed to expose and decompress the dural sac and nerve roots. For patients with bilateral symptoms, the spinous process root, the contralateral ligamentum flavum, and part of the contralateral articular process were removed to achieve bilateral decompression. Attention was paid to ensure the dural sac and nerve roots achieved adequate decompression (the neural tissue reached the conditions of blood supply improvement, recovery anatomical position recovery, and independent pulsation). The nerve roots and dura were protected while the annulus fibrosus was opened. A minimally invasive reamer was used to treat the disc.

After confirming the protected neural structures, the intervertebral disc tissues were minced using different diameters’ reamers. The nuclear material and annulus fibrosus were removed while the upper and lower endplate cartilage were scraped through a working cannula. A model case is first used to determine the appropriate case size. Autogenous and allogeneic bone was implanted into the intervertebral space through the working cannula, and the titanium expandable cage was inserted into the bone graft site. The cage was placed nearly in the middle of the intervertebral space and was confirmed by the C-arm fluoroscope. The dural sac and nerve roots were ensured to exist outside of the working cannula before placing cage, and would be re-checked after completing cage placement.

Four small longitudinal incisions were made from the marked pedicle projection. The skin, subcutaneous tissue, and deep fascia were incised successively, and the muscles were passively separated. The pedicle screws with appropriate diameter and length were inserted percutaneously under the guidance of the C-arm fluoroscope. The incision was repeatedly irrigated and checked for active bleeding before the incision was closed. After the screw and cage position was judged satisfactory, the incisions were sutured directly.

#### MIS-TLIF (L4/5 Segment)

After anesthesia, the procedure was performed on the prone. A skin incision of 3 cm to 2–3 cm lateral to the midline is made after determining the operative level and marking the skin with a C-arm image. Through this incision, a tubular retractor system was placed. The lamina, facet joint, and transverse process were exposed through a working retractor. The procedures were undergone under direct visualization rather than endoscopic visualization. The inferior and superior articular processes, ligamentum flavum, and part of the vertebral lamina were removed to expose the ipsilateral nerve root and dural sac. After extensive decompression, a discectomy was performed to remove the nuclear material and annulus fibrosus in Kambin’s triangle. If there were contralateral symptoms, contralateral decompression was also performed on cutting of the spinous process root and ligamentum flavum. Progressively large dilating bougies stretched the intervertebral space. A cage was obliquely inserted into the intervertebral space after the autogenous and allogeneic bone was implanted. The procedures of the bilateral pedicle screw were similar to PE-LIF.

### Postoperative Treatment

Both groups were treated with preventive antibiotics within 24 h following the operation. The mannitol and non-steroidal drugs were used appropriately. The patients were guided to carry out lower limb activities and low back muscle training in bed within 24 h after the operation. They started the out-of-bed movement two days post-operation. The patients were reminded to perform regular life under the protection of a brace within three months after the operation.

### Outcome Measures

The perioperative factors involving the operative time, fluoroscopy time, estimated blood loss, bed rest time, length of hospital stays, and complication rate were obtained. Patient-reported outcomes (PROs) questionnaires were administered preoperatively at three days, three months, six months, and last follow-up postoperatively, including VAS, Oswestry Disability Index (ODI). The modified MacNab standard evaluation was calculated at the last follow-up. Radiologic outcomes included intervertebral fusion rates assessed with the Bidwell evaluation criterion at the last follow-up (11).

### Statistical Analysis

All data were analyzed using IBM SPSS Version 26 (IBM Corporation, Armonk, New York, USA). The independent sample t-test was applied to compare the continuous data, which complies with the normal distribution between the two groups. Those non-normal distribution variables were analyzed by Mann-Whitney U test. We used the Chi-Square test or Fisher’s exact test to compare categorical data. Statistical significance was defined as *P *< 0.05 for all analyses.

## Result

### Baseline Characteristics and Clinical Outcomes

Eighty-nine patients were qualified for the study. The 41 patients (15 men and 26 women) who underwent the PE-LIF had a mean age of 61.85 ± 10.45 years old. The 48 patients (18 men and 30 women) who underwent MIS-TLIF had a mean age of 62.98 ± 10.52 years. The mean follow-up period was 14.13 ± 3.91 months in the PE-LIF group and 13.66 ± 3.67 months in the MIS-TLIF group. There was no significant difference between the 2 groups in terms of gender, age, body mass index, follow-up period, and surgery levels (*P *> 0.05). Demographics and baseline characteristics of the two groups are presented in Table 1.

**Table 1:** Demographics and baseline characteristics of the two groups: PE-LIF versus MIS-TLIF.

Variable	PE-LIF	MIS-TLIF	*P-*value
No. of patient	41	48	
Gender			0.929
Male	15	18	
Female	26	30	
Age (years) (mean ± SD)	61.85 ± 10.45	62.98 ± 10.52	0.531
BMI (kg/m^2^) (mean ± SD)	25.11 ± 2.58	24.47 ± 2.45	0.231
Follow-up time (months) (mean ± SD)	14.13 ± 3.91	13.66 ± 3.67	0.558
Levels of surgery			0.91
L3/4	3	1	
L4/5	24	33	
L5/S1	14	14	
Operative time (minutes) (mean ± SD)	193.41 ± 28.42	167.33 ± 28.91	<0.001*
Fluoroscopy time	40.32 ± 4.17	25.38 ± 3.58	<0.001*
Estimated blood loss (mL)	122.24 ± 18.29	157.90 ± 28.61	<0.001*
Bed rest time (hours)	39.80 ± 6.65	43.46 ± 6.28	0.009*
Hospital stays (days)	8.87 ± 1.64	9.38 ± 1.88	0.179
Complications	1	2	0.467

* *Statistically significant.*

Compared with the MIS-TLIF group, the PE-LIF group had a significantly longer operative time (193.41 ± 28.42 vs. 167.33 ± 28.91 min, *P *< 0.001), greater fluoroscopy time (40.32 ± 4.17 vs. 25.38 ± 3.58, *P *< 0.001), lower estimated blood loss (122.24 ± 18.29 vs. 157.90 ± 28.61 mL), and shorter bed rest time (39.80 ± 6.65 vs. 43.46 ± 6.28 h, *P *< 0.05). The length of hospital stays was similar between the PE-LIF group and the MIS-TLIF group (8.87 ± 1.64 vs. 9.38 ± 1.88 h, *P *> 0.05) (Table 1).

### Therapeutic Evaluation

**Figure 1:**
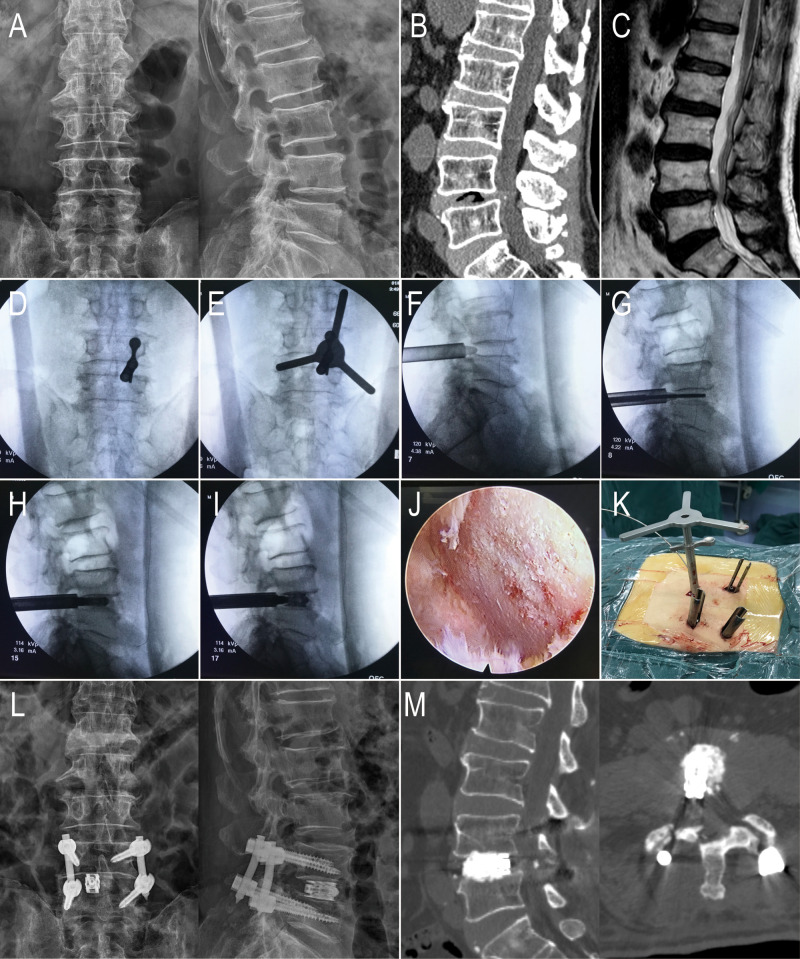
An 81-year-old male with L4-5 LSS in the PE-LIF group. (**A–C**) Preoperative X-ray, CT, and MRI showed that L4 and L5 vertebra body and the intervertebral; (**D**) The puncture needle was placed; (**E**) Using the circular saw to remove a part of the facet joint; (**F**) the working cannula were placed percutaneously; (**G,H**) using reamers of different diameters to mince the intervertebral disc tissues and conduct endplate preparation; (**I**) the titanium expandable cage was placed through the working cannula; (**J**) Decompression of the nerve root and handling the endplates under endoscopic vision (**K**) Direct vision of the working channel and the circular saw. (L-M) X-ray and CT showed the percutaneous pedicle screw fixation and the titanium expandable cage.

**Figure 2:**
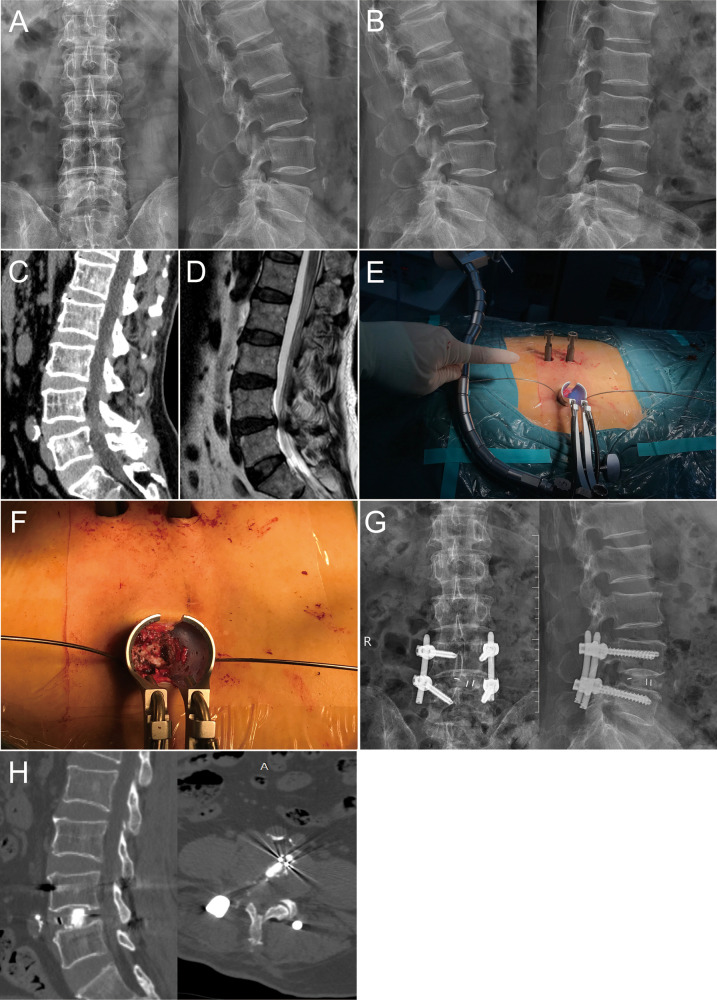
A 68-year-old male with L4-5 LSS in the MIS-TLIF group. (**A–D**) Preoperative X-ray, CT, and MRI showed the condition of the symptomatic segment, and the dynamic flexion-extension radiographs showed L4 instability; (**E**) The tubular retractor system was placed; (**F**) Decompression of nerve root and dural sac were performed under direct visualization; (**G,H**) X-ray and CT showed the posterior instrument and the cage was appropriate.

Both groups showed significant improvements in the VAS for back pain (PE-LIF: 6.46 ± 1.14 to 1.37 ± 0.66; MIS-TLIF: 6.75 ± 0.93 to 1.40 ± 0.54) and leg pain (PE-LIF: 7.83 ± 0.92 to 0.98 ± 0.61; MIS-TLIF: 7.58 ± 0.85 to 0.90 ± 0.59) (*P *< 0.001). The ODI score also significantly improved at the last follow-up after the operation (PE-LIF: 56.32 ± 9.54 to 15.32 ± 3.05; MIS-TLIF: 57.96 ± 6.92 to 14.35 ± 2.91). The VAS for both back and leg pain, and ODI scores were similar between the two groups preoperatively and at 3-month, 6-month, and the last follow-up after surgery. However, comparing the three-day postoperative data, the VAS score for back pain in the PE-LIF group was lower than that in the MIS-TLIF group with significant differences (2.55 ± 0.75 vs. 3.18 ± 0.67, *P *< 0.05) (Table 2). Following the modified Macnab standard of evaluation, the excellent or good rate was 95.12% in the PE-LIF group and 95.83% in the MIS-TLIF group at the last follow-up (*P *> 0.05) (Table 2). According to the Bridwell grading system, fusion grades in the PE-LIF group were 73.17% (*n* = 30) for grade I and 26.83% (*n* = 11) for grade II. In the MIS-TLIF group, fusion grades were 75.00% (*n* = 36) for grade I and 25.00% (*n* = 12) for grade II. There were no significant differences between the two groups in intervertebral fusion rates (*P* = 0.844). The representative cases are shown in Figures 1, 2.

**Table 2:** Preoperative and postoperative visual analogue scale (VAS), Oswestry disability index (ODI) scores and Modified MacNab (mean ± SD).

	PE-LIF	MIS-TLIF	*P-*value
VAS (back)			
Preoperative	6.46 ± 1.14	6.75 ± 0.93	0.196
Postoperative 3 days	3.10 ± 0.70	3.48 ± 0.88	0.025*
Postoperative 3 months	2.37 ± 0.77	2.40 ± 0.82	0.86
Postoperative 6 months	1.54 ± 0.60	1.71 ± 0.58	0.173
Last follow-up	1.37 ± 0.66	1.40 ± 0.54	0.814
* P* value (last vs. pre)	<0.001*	<0.001*	
VAS (leg)			
Preoperative	7.83 ± 0.92	7.58 ± 0.85	0.193
Postoperative 3 days	3.78 ± 0.76	4.02 ± 0.79	0.147
Postoperative 3 months	2.46 ± 0.64	2.33 ± 0.66	0.35
Postoperative 6 months	1.68 ± 0.61	1.71 ± 0.54	0.836
Last follow-up	0.98 ± 0.61	0.90 ± 0.59	0.534
*P* value (last vs. pre)	<0.001*	<0.001*	
ODI index			
Preoperative	56.32 ± 9.54	57.96 ± 6.92	0.351
Postoperative 3 days	32.54 ± 4.70	34.13 ± 5.13	0.134
Postoperative 3 months	25.12 ± 3.69	26.17 ± 3.99	0.206
Postoperative 6 months	20.68 ± 2.43	21.13 ± 2.47	0.399
Last follow-up	15.32 ± 3.05	14.35 ± 2.91	0.132
*P* value (last vs. pre)	<0.001*	<0.001*	
Modified MacNab			0.872
Excellence	26	37	
Good	13	9	
Fair	2	1	
Poor	0	1	
Excellence/good rate (%)	95.12	95.83	

* *Statistically significant.*

### Related Complications

There was one case of transient ankle dorsiflexion weakness in the PE-LIF group and, one case of superficial infection, one case of postoperative epidural hematoma in the MIS-TLIF group. No significant differences were seen in the complications between both groups (*P *> 0.05) (Table 1). All patients recovered without major complications such as significant vessel injury, peritoneal injury, and pulmonary embolism. No patient required revision surgery during the follow-up period.

## Discussion

LSS is defined as a degenerative condition always accompanied by loss of intervertebral disc height, degenerative lumbar spondylolisthesis, thickening of ligamentum flavum, and facet joint hypertrophy with aging, causing the spinal neurovascular structures to compressed (1). It may occur on a congenital (developmental) narrow lumbar canal, degenerative processes, or both. Neurogenic claudication is the most typical clinical feature of LSS, which is always required to be distinguished from vascular claudication (4). To date, no clear gold-standard criteria have been established to diagnose LSS. Clinicians need to integrate the combination of age, symptoms, physical examinations, and imaging findings before making medical decisions (12). The symptomatic LSS has limited patients’ daily activities and decreased their quality of life. It was reported that the costs of LSS surgeries were estimated at nearly $1.65 billion in 2007 in the United States, which placed a substantial economic burden on the medical system (13).

Traditional surgical techniques of decompression plus fusion (P/TLIF) have been widely accepted. MIS has gotten the attention of surgeons. The MIS-TLIF technique has been widely applied, which is performed under the working channel using the tubular retractor (14). Previous studies suggest that MIS-TLIF achieves satisfactory relief of symptoms in treating various degenerative lumbar diseases and can lessen tissue trauma, reduce postoperative pain, shorten hospital stays, and allow faster recovery (9, 15). Wong et al. provided evidence that MIS-TLIF was found to have a statistically significant reduction in the lower rate of reoperations and deep wound infection than open TLIF (16). In addition, a meta-analysis from Ray et al. reported that fusion rates for MIS-TLIF and open TLIF were similar and relatively high (17).

In the last decades, percutaneous endoscopic lumbar discectomy (PELD) has undergone significant development in managing lumbar degenerative diseases. The operative approach avoided largely removing the lamina, ligament flavum, or facet joints, which maintained the stability of the surgical segment. Benefiting from the minimal trauma, patients undergoing PELD have often experienced shorter bed rest and hospitalization time, and early return to work (18). However, PELD also had some downsides. This technique requires surgeons to develop skill proficiency in endoscopic spine surgery. Some scholars still questioned its incomplete removal of the disc and high incidence of revisions (19).

Based on the theory of PELD, recently developed techniques of PE-LIF achieved important minimal invasive goals. Theoretically, PE-LIF requires a smaller skin incision with less muscle dilation than other lumbar interbody fusion procedures. Osman et al. firstly applied this technique to patients with lumbar degenerative diseases in 2012 (20). The follow-up results indicated that the overall outcomes were satisfying, but there was a high complication rate. With the innovation of relevant surgical instruments and the increased technical proficiency of surgeons, more promising clinical outcomes with fewer complications were reported in recent literature (10, 21). In seven cases, Yang JC et al. (22) applied this surgical method for L4/5 single-segment LSS. There were significant improvements in symptoms for all patients, and no serious complications occurred during follow-up. A prospective cohort study by Ao et al. (23) demonstrated no significant differences in medium-short term surgical outcomes between PE-LIF and MIS-TLIF (e.g., the VAS scores, the ODI scores, the fusion rates, and complications). In fact, on average, patients of PE-LIF had faster functional recovery. A meta-analysis from Kou et al. (24) provided further evidence that the PE-LIF had advantages in terms of less intraoperative blood loss and shorter hospital stay.

For clinical outcomes based on this retrospective cohort study including 89 patients with LSS treated by PE-LIF and MIS-TLIF, it revealed no difference in clinical efficacy and safety (involving pain intensity, ODI scores, fusion rates, and complications) at the last follow postoperatively. Both groups were of satisfactory outcomes for LSS without any significant complications. The results suggested that PE-LIF presents significantly lower estimated blood loss and a shorter bed rest time than MIS-TLIF. PE-LIF provided significantly better lower back pain relief in the immediate postoperative period than MIS-TLIF. However, the cohort of patients who underwent PE-LIF appears to experience considerably more fluoroscopy times and longer operative times than the MIS-TLIF group. Previous reports showed that PE-LIF had significantly lower hospital stays than MIS-TLIF (10, 23). In this study, the length of hospital stays trended towards being lower in the PE-LIF group, but the difference was not significant (*P *= 0.179). More high-level clinical evidence should be explored.

The diameter of the single hole endoscopic channel of PE-LIF is shorter than the tubular retractor system of MIS-TLIF, which theoretically reduces tissue trauma. Moreover, a significant additional advantage of PE-LIF is that it can be operated under endoscopy. Therefore, some scholars considered that PE-LIF could reach precise decompression of the nerves and reduce the destruction of bony structures such as the articular processes or lamina, which remarkably reserve the stability of the posterior lumbar column (25). It was thought that less traumatic operation helped to restore low back muscle function, reduce the incidence of postoperative residual back pain, and allow patients to move around early while reducing bed-rest complications (26). Comparing the two groups of patients in this study cohort showed that patients in the PE-LIF group had a better early recovery than MIS-TLIF.

Despite these potential advantages, it remained unclear if PE-LIF had advantages for managing intervertebral space. Some scholars believe that with the endoscopic surgical technique, surgeons can handle the endplates under direct vision and determine adequate cartilage endplate removal (27). This may theoretically promote interbody fusion and reduce the risk of cage collapse. However, some studies also conclude that PE-LIF is prone to inadequate treatment of the cartilaginous endplate, leading to complications involving cage displacement and pseudarthrosis formation (28). In this study, compared with MIS-TLIF, PE-LIF was of similar good clinical outcomes for fusion rates, without cage displacement or collapse at the last follow-up. In our experience, PE-LIF was perhaps less efficient in treating intervertebral discs, which led to the prolongation of operation time.

Almost studies have reported the appliance of interbody implant cage. Previous studies have mainly focused on nano-hydroxyapatite/polyamide-66 Cage (n-HA/PA66) and Polyetheretherketone (PEEK), which have been widely recognized. Recent studies have suggested that the titanium expandable cage can reduce nerve roots injury, restore lumbar lordosis, and achieve indirect decompression of the spinal canal and intervertebral foramen (22, 27). However, the potential complications of bone endplate injury and pseudarthrosis could not be ignored during these procedures.

Some drawbacks deserve to be pointed out about PE-LIF. One concern for PE-LIF is the risk of increasing the ionizing radiation exposure for both patients and surgeons. The locations of the operation area and the pedicle placement were mainly confirmed by C-arm fluoroscopy rather than direct vision. Although not directly addressed in our research, repeat fluoroscopy could potentially increase the operation time. On the other hand, PE-LIF requires significant time to improve the learning curve. Surgeons should strictly grasp the indications and be familiar with percutaneous endoscopy and percutaneous pedicle screw placement techniques. Electromyography monitoring is recommended for avoiding potential serious complications, including nerve root injury and dural tears (29).

The present study had several limitations. Firstly, this was a retrospective study and the sample size was relatively small. All patients included in this research were treated in a single center. Secondly, only patient with single-level LSS is recruited, which may result in selection bias. Thirdly, some postoperative radiographic parameters involving the disc height, foraminal height, and lumbar canal cross-sectional area were not reported in this study and should be investigated in future studies. Lastly, the follow-up period was relatively short for evaluating long-term effects.

## Conclusion

The present study results demonstrate that both PE-LIF and MIS-TLIF are safe and effective for LSS. PE-LIF has a definite short-term curative effect with less trauma. Nevertheless, considering the limitations, further evidence with long-term follow-up and larger sample size should be carried out to explore the differences in outcomes after PE-LIF and MIS-TLIF.

## Data Availability

The raw data supporting the conclusions of this article will be made available by the authors, without undue reservation.
